# Chlormequat Chloride Inhibits TM3 Leydig Cell Growth via Ferroptosis-Initiated Inflammation

**DOI:** 10.3390/cells13110979

**Published:** 2024-06-05

**Authors:** Xiaoxia Wang, Chenping Kang, Wanqian Guo, Haoran Zhang, Qianqian Xiao, Weidong Hao

**Affiliations:** 1 Department of Toxicology, School of Public Health, Peking University, Beijing 100191, China; xxwang@stu.pku.edu.cn (X.W.); kcp0185@pku.edu.cn (C.K.); 1710306117@pku.edu.cn (W.G.); 2211210119@stu.pku.edu.cn (H.Z.); 2 Beijing Key Laboratory of Toxicological Research and Risk Assessment for Food Safety, Beijing 100191, China

**Keywords:** chlormequat chloride, Leydig cells, ferroptosis, inflammation, ferritin light chain, regulatory cell death

## Abstract

Ferroptosis hallmarked by lipid peroxidation and iron homeostasis imbalance is involved in the occurrence and development of various diseases. The plant growth regulator chlormequat chloride (CCC) can contribute to the causality and exacerbation of reproductive disorders. However, the mechanism by which CCC may cause Leydig cell attenuation remains poorly understood. In this study, TM3 Leydig cells were used to investigate the inhibitory effect of CCC on cell growth and its possible mechanism. The results showed that CCC caused apoptosis, pyroptosis, ferroptosis and necroinflammation in TM3 cells. By comparing the effects of ferroptosis inhibitor Ferrostatin-1 (Fer-1) and pan-Caspase inhibitor Z-VAD-FMK (ZVF) on lipid peroxidation and Caspase-mediated regulated cell death (RCD), we found that Fer-1 was better at rescuing the growth of TM3 cells than ZVF. Although ZVF reduced mitochondrial ROS level and inhibited the activation of Caspase3 and Caspase1, it could not significantly ameliorate lipid peroxidation and the levels of IL-1β and HMGB1 like Fer-1. Therefore, ferroptosis might be a key non apoptotic RCD mode responsible for CCC-driven inflammation, leading to weakened viability and proliferation of TM3 cells. In addition, overexpression of ferritin light chain (FTL) promoted the resistance of TM3 cells to CCC-induced ferroptosis-mediated inflammation and to some extent improved the inhibition of viability and proliferation. Altogether, ferroptosis-initiated inflammation might play a key role in CCC-impaired TM3 cell growth.

## 1. Introduction

Chlormequat chloride (CCC) is a kind of widely used plant growth regulator that mainly inhibits the synthesis of gibberellin, effectively controls plant elongation, and thus improves the yield and quality of agricultural products [1,2]. With the increasingly widespread application of CCC, reports on its residual issues and poisoning cases have occurred from time to time [3,4,5,6]. According to reports, CCC gives rise to male reproductive toxicity. It not only caused deterioration of semen quality, testicular damage, and decreased testosterone level in rodents [7,8,9] but also inhibited testosterone secretion in primary rat Leydig cells via endoplasmic reticulum stress [10] and induced iron overload by ferritinophagy activation contributing to lipid peroxidation in GC-1 cells [11]. However, a further mechanism has not yet been fully determined.

Normally, cells that cannot neutralize and repair toxin-induced damage undergo regulated cell death (RCD), such as apoptosis, which exhibits significant morphological changes and is activated by specific Cysteinyl aspartate-specific proteinases (Caspases) and signaling pathways [12]. However, due to its anti-inflammatory and immunosuppressive effects, apoptosis could not account for the inflammation after toxin exposure [13,14,15]. In contrast to apoptosis, regulated necrosis leads to the release of immunogenic intracellular molecules, which in turn triggers inflammation [16]. Regulated necrosis commonly includes necroptosis, pyroptosis, and ferroptosis [12]. Pyroptosis is the activation of multiple Caspases, including Caspase1, mediated by inflammasomes, causing splicing and aggregation of various Gasdermin family members, leading to cell perforation and ultimately cell death. Meanwhile, Caspase1 also processes precursor IL-1β into mature IL-1β and releases it to amplify inflammation [17,18]. Necroptosis is not regulated by Caspases but is mediated by receptor interacting protein kinase 3 (RIPK3), RIPK1, and mixed-lineage kinase domain-like protein (MLKL) and can be activated under apoptosis-deficient conditions [19,20]. Ferroptosis is characterized by deficiency of thiol-based redox regulation, accumulation of lipid peroxidation products, and disturbed iron homeostasis [13,21]. Iron homeostasis is to some extent controlled by ferritin, an iron storage molecule composed of ferritin heavy chain (FTH) and ferritin light chain (FTL) [22]. Their proportional contribution to the complex varies by tissue. In subcellular compartments, ferritin is present in the mitochondria and nucleus. Additionally, it not only stores iron but also has characteristics such as immunomodulatory attributes [23].

Currently, research is rapidly increasing on the association between inflammation caused by ferroptosis and various diseases [24,25]. In this study, with a focus on FTL, we studied the effect of CCC on inducing ferroptosis and further stimulating inflammation to suppress TM3 cell growth.

## 2. Materials and Methods

### 2.1. Cell Lines

#### 2.1.1. TM3 Leydig Cells

TM3 mouse Leydig cell line was purchased from Cell Resource Center, Peking Union Medical College (PCRC). Cells were cultured in Dulbecco’s Modified Eagle’s Medium (Gibco, Grand Island, NY, USA) supplemented with 10% fetal bovine serum (TransgenBiotech, Beijing, China) and antibiotics (100 μg/mL penicillin and 100 U/mL streptomycin) (MCE, Princeton, NJ, USA) at 37 °C in the presence of 5% CO_2_.

#### 2.1.2. TM3 Cells Overexpressing FTL

The sequence of FTL (Gene ID: 14325) was searched in NCBI database, and primers were designed and synthesized by Shanghai Bioengineering Co., Ltd. (Shanghai, China). The plasmid pMT406 was purchased from Shanghai Bioengineering Co., Ltd., and the packaging plasmids pCMV-dR8.9 and pCMV-VSV-G were purchased from Addgene (Cambridge, MA, USA).

To construct TM3 cells overexpressing FTL, FTL primers were synthesized: (forward) 5′-TGGCAAAGAATTGGATCCGCCACCATGACCTCTCAGATTCGTCAGA-3′, (reverse) 5′-CATAATACTAGTCTCGAGCTAGTCGTGCTTGAGAGTGAGG-3′. The target gene was amplified by polymerase chain reaction (PCR). Gel purification was used to isolate and purify vector DNA fragments derived from restriction endonuclease digests. The target gene was ligated into vector DNA fragments and transformed into E.coli DH5α-Competent Cells. Transformants were identified using colony PCR and the positive clones were sequenced. Plasmid extraction was performed on clones with accurate sequencing.

#### 2.1.3. TM3 Cells with FTL Knockdown

To construct TM3 cells with FTL knockdown, three short interfering RNA target primers were designed based on FTL gene sequence, and the construction framework of FTL-shRNA lentiviral vectors is shown in Table 1. The plasmid pMagic7.1 was purchased from Shanghai Bioengineering Co., Ltd., and the packaging plasmids pCMV-dR8.9 and pCMV-VSV-G were purchased from Addgene.

The single strand primers were annealed to double-stranded DNA and connected to double-enzyme tangent RNA interference vectors. The correct transformants were verified through sequencing, and high-purity plasmids were extracted.

The lentiviral vector was packaged and used to infect TM3 cells after completing viral titer assay. According to the optimal multiplicity of infection (MOI), cells were cultured in a 24-well plate to be infected with corresponding doses of virus and polybrene (Solarbio, Beijing, China) when the cell density reached 30% to 50%. TM3 cells stably transfected with corresponding plasmids were screened by puromycin (HARVEYBIO, Beijing, China). We observed good cell growth and stable expression of enhanced green fluorescence protein (GFP) under Nikon A1 laser confocal microscope (Nikon, Tokyo, Japan).

### 2.2. Cell Viability Assay

Cell Counting Kit-8 (CCK-8, APE×BIO, Houston, TX, USA) assay was performed to investigate cell viability. Briefly, cells were cultured in 96-well plates with 3000 cells per well. At the end of treatment, cells were incubated with CCK-8 at 37 °C for 1 h. The absorbance of each well was detected by a FLUOstar Omega multifunctional microplate reader (BMG LRBTECH, Offenburg, Germany) at a wavelength of 450 nm. The experiment was repeated three times.

### 2.3. Cell Cycle Assay

7-AAD (Invitrogen, Carlsbad, CA, USA) was used to detect the cell cycle. Cells were cultured in 12-well plates with 1.5 × 10^5^ cells per well. At the end of treatment, harvested cells were fixed in ice-cold 70% ethanol at 4 °C for 2 h and incubated with 7-AAD in the dark at 4 °C for 1 h according to manufacturer’s instructions. CytoFLEX Flow Cytometer (Beckman Coulter, Brea, CA, USA) was used for data collection and analysis. The experiment was repeated three times.

### 2.4. Detection of Mitochondrial ROS

MitoSOX Red (MCE, Princeton, NJ, USA) is a live cell fluorescent probe specifically targeting mitochondrial superoxide (MitoSOX) to indicate mitochondria reactive oxygen species (ROS) level. Cells were cultured in 12-well plates with 1.5 × 10^5^ cells per well. At the end of treatment, harvested cells were incubated with MitoSOx Red at 37 °C for 30 min according to manufacturer’s instructions. CytoFLEX Flow Cytometer was used for data collection and analysis. The experiment was repeated three times.

### 2.5. Detection of Cell Apoptosis

The Annexin V-PE/7-AAD assay kit (Pricella, Wuhan, China) was used to detect cell apoptosis. Specifically, cells were cultured in 12-well plates with 1.5 × 10^5^ cells per well. At the end of treatment, cells were incubated with Annexin V-PE and 7-AAD at room temperature in the dark for 15 min according to manufacturer’s instructions. CytoFLEX Flow Cytometer was used for data collection and analysis. The experiment was repeated three times.

### 2.6. Detection of Lipid Peroxidation

C11-BODIPY581/591 (MCE, Princeton, NJ, USA) is an oxidation-sensitive fluorophore whose maximal ex/em wavelengths shift from 581/591 nm to 488/510 upon lipid peroxidation and is used to indicate antioxidant properties in living cells [26]. According to manufacturer’s instructions, at the end of treatment, cells were incubated with C11-BODIPY581/591 (2 μM) for 1 h at 37 °C. Nikon A1 laser confocal microscope or CytoFLEX Flow Cytometer was used for data collection and analysis. The ratio of mean fluorescence intensity (MFI) between oxidized C11-BODIPY488/510 (oxC11-BODIPY) and unoxidized C11-BODIPY581/591 (unoxC11-BODIPY) was calculated to indicate the level of lipid peroxidation. The experiment was repeated three times.

### 2.7. Detection of Fe^2+^

FerroOrange (Dojindo, Beijing, China) was used to target ferrous iron in living cells. According to manufacturer’s instructions, cells were incubated with phosphate-buffered saline (PBS) containing 1 μM FerroOrange and 1 μM ER-Tracker Green (Yeasen, Shanghai, China) at 37 °C for 30 min, followed by detection using Nikon A1 laser confocal microscope or CytoFLEX Flow Cytometer. The experiment was repeated three times.

### 2.8. CFSE Staining

Carboxyfluorescein succinimidyl ester (CFSE, Invitrogen, Carlsbad, CA, USA) can be uniformly inherited by the cells with cell division and proliferation, and its attenuation is proportional to the number of cell divisions. The harvested cells were adjusted to a concentration of 10^6^ cells/mL and incubated with 5 µM CFSE at 37 °C for 15 min. Aliquots of stained cells were distributed into culture plates for appropriate cultivation. CytoFLEX Flow Cytometer was used for data collection and analysis. The experiment was repeated three times.

### 2.9. Western Blotting

Cell culture dish was placed on ice and wash the cells with ice-cold PBS. PBS was aspirated and then ice-cold RIPA lysis buffer (Beyotime, Shanghai, China) or nuclear and cytoplasmic protein extraction reagents (Biosharp, Guangzhou, China) with protease and phosphatase inhibitor cocktail (Beyotime, Shanghai, China) were added. Adherent cells were scraped off and sonicated using VirTis Virsonic 100 ultrasonic cell disrupter (SP industries Inc., Warminster, PA, USA). Cell lysate was centrifuged (15,000× *g*) at 4 °C for 15 min. The Pierce™ BCA Protein Assay Kit (Thermo Scientific, Waltham, MA, USA) was used for quantitative determination of protein concentration in supernatant. An equal amount of total protein was separated by sodium dodecyl sulfate polyacrylamide gel electrophoresis (SDS-PAGE) and was transferred onto a nitrocellulose membrane (Pall Corporation, Ann Arbor, MI, USA). Membrane was blocked with 5% non-fat milk for 2 h at room temperature and was incubated with appropriate dilutions of primary antibodies overnight in 5% BSA buffer at 4 °C, including anti-β-actin (Transgen Biotech, Beijing, China), anti-β-tubulin (Abmart, Shanghai, China), anti-GAPDH (Proteintech, Hubei, China), anti-Histone H3 (CST, Danvers, MA, USA), anti-FTL (Abmart, Shanghai, China), anti-Caspase1 (Proteintech, Hubei, China), anti- Caspase3 (Abmart, Shanghai, China), anti-p-P53 (Abmart, Shanghai, China), anti-P53 (Proteintech, Hubei, China), anti-NLRP3 (Abcam, Cambridge, MA, USA), anti-GPX4 (Abmart, Shanghai, China), anti-HMGB1 (Abmart, Shanghai, China), anti-TXNIP (Abmart, Shanghai, China), anti-TfR (Abmart, Shanghai, China), anti-FACL4 (Abmart, Shanghai, China), anti-xCT (Abmart, Shanghai, China), anti-IL-1β (Abclonal, Wuhan, China), anti-GSDMD (Abclonal, Wuhan, China), and anti-NOCA4 (Abclonal, Wuhan, China). Membrane was washed three times in Tris-Buffered Saline with Tween^®^ 20 (TBST), 5 min each wash, and incubated with the recommended dilution of HRP secondary antibodies (Gene-Protein Link Biotech, Beijing, China) in TBST at room temperature for 2 h. Immunoreactive bands were visualized using Super ECL Kit (Biodragon, Beijing, China) and Uvitec Cambridge Chemiluminescence Imaging System (Uvitec, Cambridge, UK).

### 2.10. Immunofluorescence

Cells were placed on coverslips at an appropriate dilution to grow until they reached the desired confluence. Then, they were treated with different concentrations of CCC for 24 h, and necroptosis inducer TSZ (APExBio, Houston, TX, USA), a combination of TNF-alpha (T), Smac mimetics (S), and z-VAD (Z), was used to treat TM3 cells for 12 h as a positive control. Cells were fixed with 4% formaldehyde for 15 min at room temperature and treated with 0.3% Triton X-100 for 5 min to result in permeabilized cell preparations. Samples were blocked in 5% normal goat serum (Beyotime, Shanghai, China) with 1% Triton X-100 (PBST) for 1 h and incubated overnight with diluted primary antibodies at 4 °C with gentle agitation, including anti-p-MLKL (CST, Danvers, MA, USA), anti-p-RIP3 (CST, Danvers, MA, USA), and anti-β-tubulin (Abmart, Shanghai, China). Then, cells were incubated with fluorochrome-conjugated secondary antibodies (Proteintech, Hubei, China) diluted in PBST for 2 h at room temperature in the dark. Coverslips were mounted and examined using Nikon A1 laser confocal microscope.

### 2.11. Statistical Analysis

The data were analyzed using SPSS 24.0 and expressed as mean ± standard deviation (mean ± SD). Comparison among multiple groups was conducted using one-way analysis of variance (ANOVA). When comparing between two groups, the LSD test was used for data with homogeneous variances, and Dunnett’s T3 test was used for data with heterogeneous variances. A value of *p* < 0.05 indicated a statistically significant difference.

## 3. Results

### 3.1. CCC Inhibited the Viability and Proliferation of TM3 Cells

**Figure 1:**
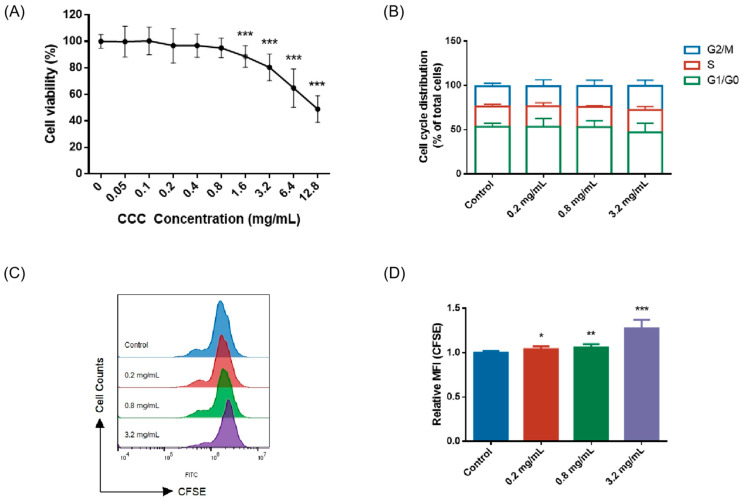
The effects of CCC on the viability, cycle, and proliferation of TM3 cells. (**A**) The percentage of surviving TM3 cells after exposure to different concentrations of CCC for 24 h; *n* = 8. (**B**) Representative data of cell cycle; *n* = 3. (**C**,**D**) Representative data of CFSE staining and its relative mean fluorescence intensity (MFI); *n* = 3. All data are expressed as mean ± SD. Statistical significance was tested by one-way ANOVA; * *p* < 0.05 vs. the control group, ** *p* < 0.01 vs. the control group, and *** *p* < 0.001 vs. the control group. ANOVA, analysis of variance.

TM3 cells were treated with 0, 0.05, 0.1, 0.2, 0.4, 0.8, 1.6, 3.2, 6.4, and 12.8 mg/mL of CCC for 24 h and the cell viability was measured. The results showed that cell viability decreased to 80% after treatment with 3.2 mg/mL CCC for 24 h (Figure 1A). Therefore, three CCC concentrations were used for subsequent experiments, namely 0.2 mg/mL, 0.8 mg/mL, and 3.2 mg/mL. As shown in Figure 1B, CCC had no effect on the cell cycle but significantly inhibited cell proliferation (Figure 1C,D).

### 3.2. CCC Caused Ferroptosis, Pyroptosis, Apoptosis, and Necroinflammation in TM3 Cells

**Figure 2:**
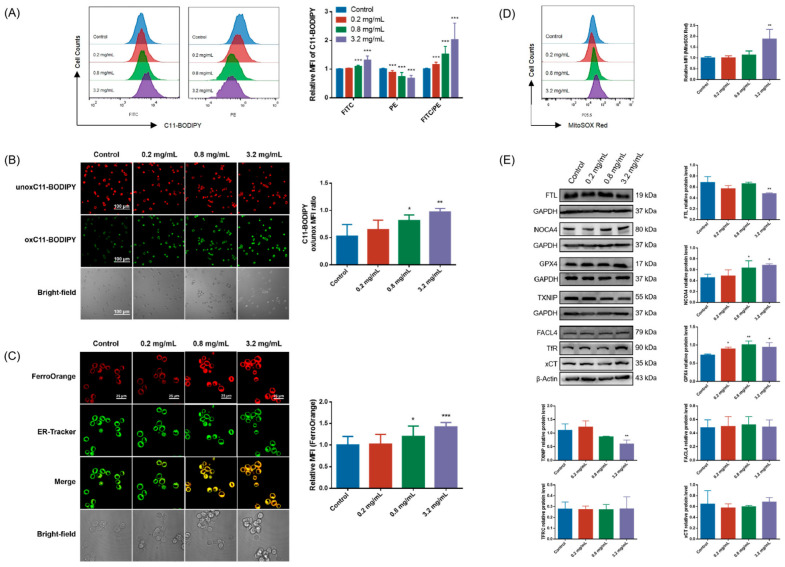
CCC-triggered ferroptosis in TM3 cells. (**A**) Representative data and relative MFI of C11-BODIPY staining detected by flow cytometry. Oxidized C11-BODIPY488/510-positive cells: FITC filter; unoxidized C11-BODIPY581/591-positive cells: PE filter; *n* = 3. (**B**) Representative fluorescence images of TM3 cells stained with C11-BODIPY (red—unoxidized C11-BODIPY581/591-positive cells, green—oxidized C11-BODIPY488/510-positive cells) (40×, scale bar = 100 μm) and the MFI ratio of oxidized to unoxidized C11-BODIPY; *n* = 3. (**C**) Representative fluorescence images of TM3 cells stained with FerroOrange (red—FerroOrange, green—ER-Tracker) (100×, scale bar = 25 μm) and their relative MFI; *n* = 3. (**D**) Representative data of MitoSOX Red staining and its relative MFI; *n* = 3. (**E**) Representative Western blots of FTL, NOCA4, GPX4, TXNIP, FACL4, TfR, and xCT, as well as semi-quantitative analysis of protein expression calculating the relative expression of each protein using target protein/internal reference protein; *n* = 3. All data are expressed as mean ± SD. Statistical significance was tested by one-way ANOVA; * *p* < 0.05 vs. the control group, ** *p* < 0.01 vs. the control group, and *** *p* < 0.001 vs. the control group. ANOVA, analysis of variance.

Lipid peroxidation, mitochondrial ROS level, and intracellular Fe^2+^ content were measured as the indicators of ferroptosis. Flow cytometry was used to detect at the whole cell level that CCC reduced the MFI of unoxidized C11-BODIPY581/591 (PE) while increasing the MFI of oxidized C11-BDIPY488/510 (FITC) and the MFI ratio of oxidized to unoxidized C11-BODIPY (Figure 2A). High-specificity visualization observation of cells was performed using laser confocal microscopy, and quantitative analysis results show that CCC treatment enhanced the MFI ratio of oxidized and unoxidized C11-BODIPY (Figure 2B). Mitochondrial ROS level and intracellular Fe^2+^ content also showed a rising trend in CCC-treated cells (Figure 2C,D). In addition, the results of detecting ferroptosis-related proteins from whole cell lysates showed that CCC did not alter the protein levels of FACL4, TfR, and xCT (Figure 2E). But, it reduced the protein levels of FTL and TXNIP and amplified the expression of NOCA4 and GPX4 (Figure 2E).

It has been reported that TXNIP downregulation can activate P53, a marker of the DNA damage response (DDR) [27], and P53 activation may regulate ferroptosis through various mechanisms [28,29]. So, we detected the expression of P53 and p-P53 in the nucleus, cytoplasm, and whole-cell lysate, and no changes were detected in TM3 cells exposed to CCC (Figure S1).

**Figure 3:**
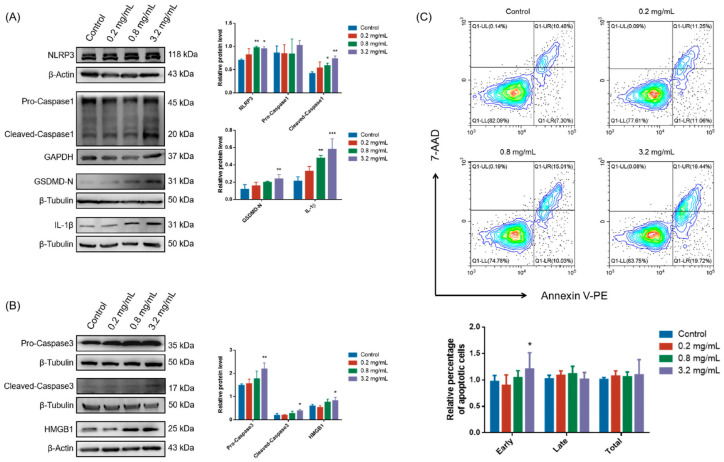
CCC-induced pyroptosis, apoptosis, and necroinflammation in TM3 cells. (**A**,**B**) Representative Western blots of NLRP3, Caspase1, GSDMD-N, IL-1β, Caspase3, and HMGB1, as well as semi-quantitative analysis of protein expression calculating the relative expression of each protein using target protein/internal reference protein; *n* = 3. (**C**) Representative flow cytometry contour plots of TM3 cells treated with CCC for 24 h stained with PE-conjugated Annexin V/7-AAD. The percentage of cells in each quadrant (Q1-UL, necrotic cells; Q1-UR, late apoptotic cells; Q1-LL, live cells; Q1-LR, early apoptotic cells) is indicated. The relative percentage of apoptotic cells was the ratio of the percentage of apoptotic cells in the CCC treatment group with different concentrations to the percentage of apoptotic cells in the control group. All data are expressed as mean ± SD. Statistical significance was tested by one-way ANOVA; * *p* < 0.05 vs. the control group, ** *p* < 0.01 vs. the control group, and *** *p* < 0.001 vs. the control group. ANOVA, analysis of variance.

Although excessive lipid peroxidation is crucial for the execution of ferroptosis, it is also associated with cell apoptosis, necroptosis, and pyroptosis to a certain extent. We investigated whether these regulated cell death patterns occurred in TM3 cells treated with CCC. No changes in the expression of p-RIPK3 or p-MLKL were detected (Figure S2), indicating that necroptosis might not be involved in CCC-triggered TM3 death. But, we found that CCC increased the expression of NLRP3, cleaved-Caspase1, GSDMD-N, and IL-1β (Figure 3A), as well as promoted the relative percentage of early apoptotic cells and the expression of Caspase3 and cleaved-Caspase3 in TM3 cells (Figure 3B,C). These results indicated that after CCC exposure, most of the reduced TM3 might undergo Caspase- or lipid peroxidation-dependent RCD, particularly involving mechanisms of cell apoptosis, pyroptosis, and ferroptosis rather than necroptosis.

Further, we examined the release of high-mobility group box-1 protein (HMGB1), which is a typical damage-associated molecular pattern (DAMP) signal and a common marker of necroinflammation. We found that CCC triggered the release of HMGB1 in TM3 cells (Figure 3B).

### 3.3. Ferroptosis Inhibitor and Pan-Caspase Inhibitor Improved CCC-Inhibited Cell Viability and Proliferation by Suppressing Ferroptosis, Pyroptosis, or Apoptosis

**Figure 4:**
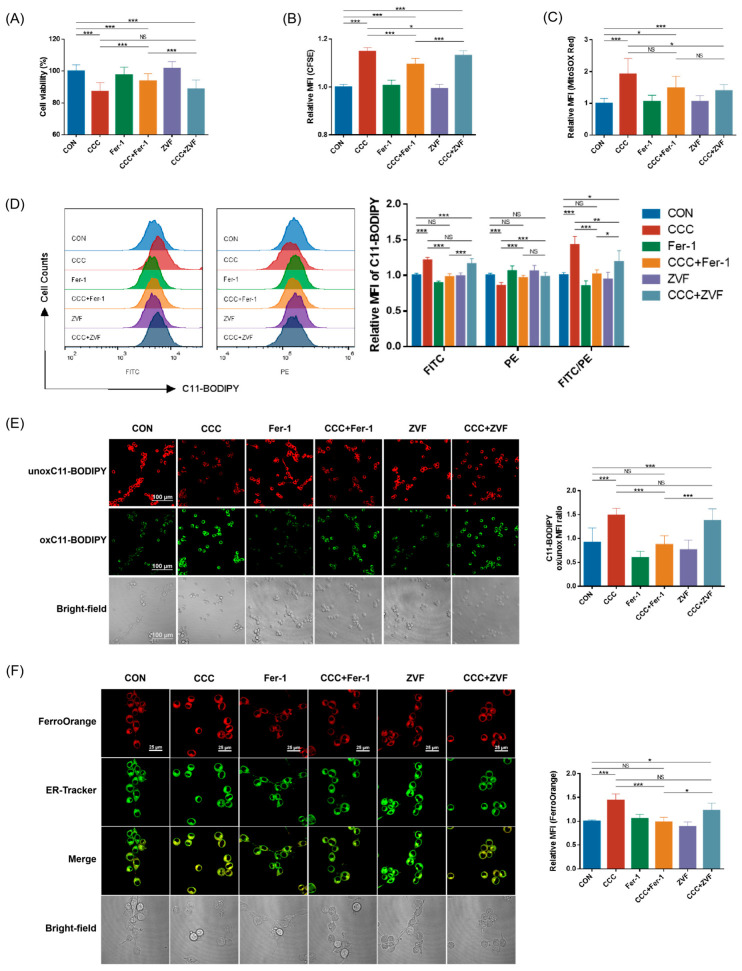
Protective effects of Fer-1 and ZVF on TM3 cells treated with CCC. (**A**) The percentage of surviving TM3 cells after exposure to CCC (3.2 mg/mL) with or without Fer-1 (10 μM) or ZVF (10 μM) for 24 h; *n* = 8. (**B**) Relative MFI of CFSE staining; *n* = 4. (**C**) Relative MFI of MitoSOX Red staining; *n* = 4. (**D**) Representative data and relative MFI of C11-BODIPY staining detected by flow cytometry. Oxidized C11-BODIPY488/510-positive cells: FITC filter; unoxidized C11-BODIPY581/591-positive cells: PE filter; *n* = 4. (**E**) Representative fluorescence images of TM3 cells stained with C11-BODIPY (red—unoxidized C11-BODIPY581/591-positive cells, green—oxidized C11-BODIPY488/510-positive cells) (40×, scale bar = 100 μm) and the MFI ratio of oxidized to unoxidized C11-BODIPY; *n* = 3. (**F**) Representative fluorescence images of TM3 cells stained with FerroOrange (red—FerroOrange, green—ER-Tracker) (100×, scale bar = 25 μm) and their relative MFI; *n* = 3. All data are expressed as mean ± SD. Statistical significance was tested by one-way ANOVA; * *p* < 0.01, ** *p* < 0.05, *** *p* < 0.001. ANOVA, analysis of variance. NS indicates no statistical significance.

To further verify the role of ferroptosis in CCC-induced cell growth inhibition, we treated cells with a synthetic antioxidant Ferrostatin-1 (Fer-1), which prevents damage to membrane lipids via a reductive mechanism, thereby inhibiting cell death. In addition, considering that CCC induced the activation of Caspase3, this suggests that apoptosis might also be involved in CCC-induced TM3 cell death. We investigated whether inhibiting apoptosis via pan-Caspase inhibitor Z-VAD-FMK (ZVF) could salvage the viability and proliferation of TM3 cells damaged by CCC (3.2 mg/mL). The results showed that only Fer-1 (10 μM) effectively ameliorated cell viability (Figure 4A), lipid peroxidation (Figure 4D,E), and intracellular Fe^2+^ content (Figure 4F), and ZVF (10 μM) alleviated the increase in mitochondrial ROS level (Figure 4C). Both Fer-1 and ZVF improved CCC-induced cell proliferation inhibition (Figure 4B), but Fer-1 had a better effect.

**Figure 5:**
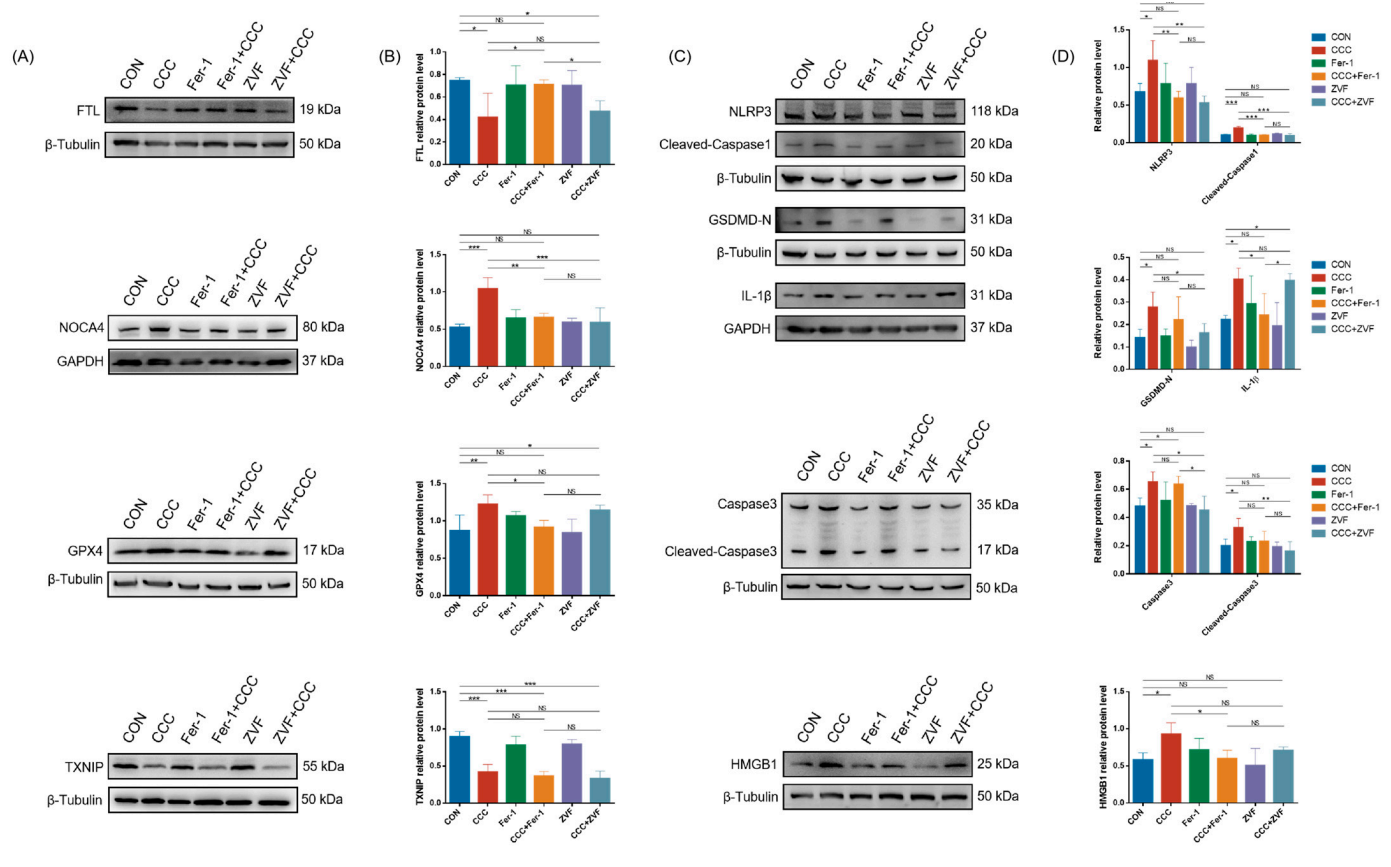
The effects of Fer-1 and ZVF on CCC-induced ferroptosis, apoptosis, and pyroptosis in TM3 cells. (**A**,**C**) Representative Western blots of FTL, NOCA4, GPX4, TXNIP NLRP3, Caspase1, GSDMD-N, IL-1β, Caspase3, and HMGB1 (*n* = 3). (**B**,**D**) Semi-quantitative analysis of protein expression calculating the relative expression of each protein using target protein/internal reference protein; *n* = 3. All data are expressed as mean ± SD. Statistical significance was tested by one-way ANOVA; * *p* < 0.01, ** *p* < 0.05, *** *p* < 0.001. ANOVA, analysis of variance. NS indicates no statistical significance.

In addition, Fer-1 rescued the expression of FTL, NOCA4, and GPX4 in TM3 cells treated with CCC, but neither Fer-1 nor ZVF improved the reduction of TXNIP (Figure 5A,B). As shown in Figure 5C,D, Fer-1 and ZVF repaired the levels of NLRP3 and cleaved-Caspase1, while only ZVF blocked the expression of GSDMD-N, Caspase3, and cleaved-Caspase3. Additionally, the levels of IL-1β and HMGB1 could be cut off by Fer-1 but not by ZVF. Therefore, the results suggested that ferroptosis might largely drive the release of danger signals from TM3 cells exposed to CCC.

### 3.4. Overexpressing FTL Overcame CCC-Damaged Cell Viability and Proliferation via Suppressing Ferroptosis

**Figure 6:**
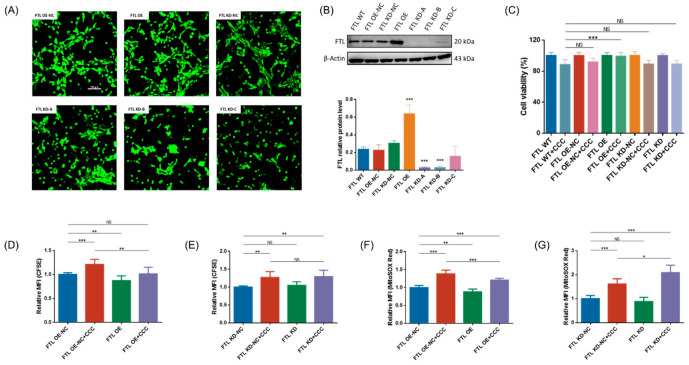
The effects of CCC on TM3 cells with changed FTL expression. (**A**) Representative fluorescence images of TM3 cells transfected with six different expression constructs (green—enhanced GFP) (20×, scale bar = 100 μm): FTL overexpressing negative control vector (FTL OE-NC), FTL overexpressing vector (FTL OE), FTL knockdown negative control vector (FTL KD-NC), and FTL knockdown vector (FTL KD-A/B/C); *n* = 3. (**B**) Representative Western blots of FTL and semi-quantitative analysis of protein expression calculating the relative expression of each protein using target protein/internal reference protein; *n* = 3. (**C**) The percentage of surviving TM3 cells with altered FTL expression after exposure to CCC for 24 h; *n* = 8. (**D**,**E**) Relative MFI of CFSE staining; *n* = 3. (**F**,**G**) Relative MFI of MitoSOX Red staining; *n* = 3. All data are expressed as mean ± SD. Statistical significance was tested by one-way ANOVA; * *p* < 0.01, ** *p* < 0.05, *** *p* < 0.001. ANOVA, analysis of variance. NS indicates no statistical significance.

FTL is the key to regulating iron homeostasis. To understand the role of FTL in CCC-induced Leydig cell damage, we next generated TM3 cells with altered FTL expression. We transfected TM3 cells with six different expression constructs: FTL overexpressing negative control vector (FTL OE-NC), FTL overexpressing vector (FTL OE), FTL knockdown negative control vector (FTL KD-NC), and FTL knockdown vector (FTL KD-A/B/C) (Figure 6A). Immunoblotting analysis was performed to demonstrate the biological functions of these constructs. The results showed that the FTL level of cells transfected with overexpressing vector was 2.8 times higher than that of cells transfected with negative control vectors, while FTL content decreased by 91%, 90%, and 48% in FTL KD-A/B/C cells, respectively (Figure 6B). Therefore, we chose FTL OE and FTL KD-A (FTL KD) for subsequent experiments.

**Figure 7:**
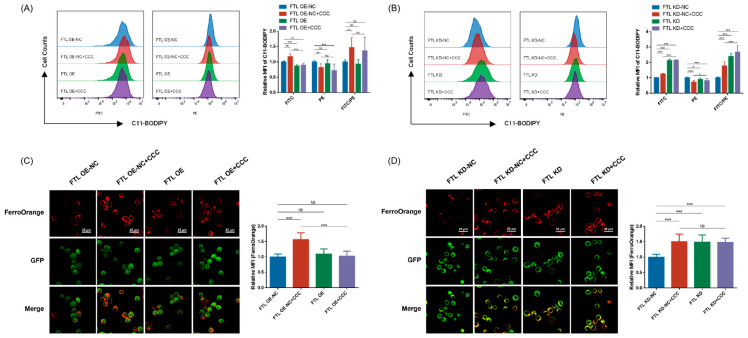
Lipid peroxidation and Fe^2+^ level in CCC treated TM3 cells with changed FTL expression. (**A**,**B**) Representative data on C11-BODIPY staining and its relative MFI ratio in TM3 cells with overexpressing or knockdown of FTL; *n* = 3. (**C**,**D**) Representative fluorescence images of TM3 cells stained with FerroOrange (red—FerroOrange, green—enhanced GFP) (100×, scale bar = 25 μm) and their relative MFI; *n* = 3. All data are expressed as mean ± SD. Statistical significance was tested by one-way ANOVA; * *p* < 0.01, ** *p* < 0.05, *** *p* < 0.001. ANOVA, analysis of variance. NS indicates no statistical significance.

The viability was detected with CCK8 in TM3 cells transfected with the aforementioned vector and treated with CCC. The results showed that FTL OE were less sensitive to CCC compared to untransfected cells (FTL WT) (Figure 6C). Also, we tested whether overexpressing FTL could overcome CCC-induced ferroptosis. It was shown that FTL overexpression could rescue CCC-induced proliferation inhibition and the increased mitochondrial ROS level (Figure 6D,F). The proliferation of FTL OE increased compared to FTL OE-NC, and FTL KD+CCC showed higher mitochondrial ROS level compared to FTL KD-NC+CCC. In addition, the lipid peroxidation was solely in part rescued, while Fe^2+^ was almost fully rescued in FTL OE (Figure 7A,C). FTL-KD exhibited higher lipid peroxidation and Fe^2+^ content compared to FTL KD-NC (Figure 7B,D).

**Figure 8:**
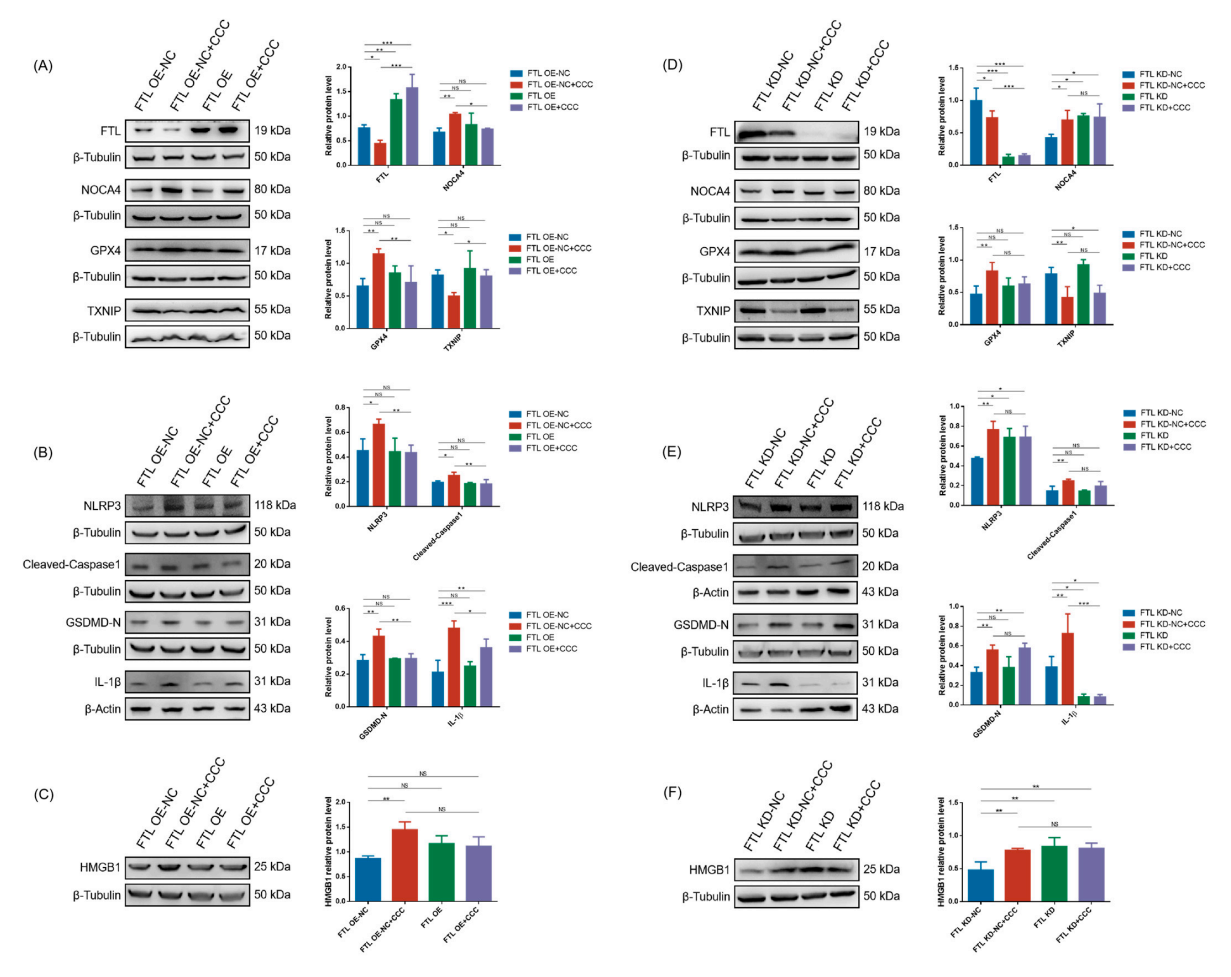
The effect of changing FTL expression on CCC-induced ferroptosis and inflammation in TM3 cells. (**A**,**D**) Representative Western blots of FTL, NCOA4, GPX4, and TXNIP in TM3 cells with FTL overexpressing or knockdown and semi-quantitative analysis of protein expression calculating the relative expression of each protein using target protein/internal reference protein; *n* = 3. (**B**,**E**) Representative Western blots of NLRP3, Caspase1, GSDMD-N, and IL-1β in TM3 cells with FTL overexpressing or knockdown and semi-quantitative analysis of protein expression calculating the relative expression of each protein using target protein/internal reference protein; *n* = 3. (**C**,**F**) Representative Western blots of HMGB1 in TM3 cells with FTL overexpressing or knockdown and semi-quantitative analysis of protein expression calculating the relative expression of HMGB1 using target protein/internal reference protein; *n* = 3. All data are expressed as mean ± SD. Statistical significance was tested by one-way ANOVA; * *p* < 0.01, ** *p* < 0.05, *** *p* < 0.001. ANOVA, analysis of variance. NS indicates no statistical significance.

Next, we tested if targeting FTL could change CCC-induced ferroptosis-related proteins. The results showed that compared to cells transferred with negative control vectors, overexpression of FTL could improve the expression of NCOA4, GPX4, and TXNIP, but they still exhibited abnormal trends in FTL knockdown cells (Figure 8A,D).

In addition, the expression of NLRP3, cleaved-Caspase1, GSDMD-N, and IL-1β in the FTL OE+CCC group was lower than that in the FTL OE NC+CCC group. There was no statistically significant difference in the expression of NLRP3, cleaved-Caspase1, and GSDMD-N between FTL KD+CCC and FTL KD-NC+CCC (Figure 8B,E). However, regardless of whether the FTL knockdown cells were treated with CCC or not, IL-1β was significantly reduced, suggesting that FTL deletion might cause immunosuppression (Figure 8B,E). And there was no statistically significant difference in HMGB1 level in TM3 cells exposed to CCC with altered FTL expression compared to cells transfected with negative control vectors (Figure 8C,F).

## 4. Discussion

In this study, we reported that CCC inhibited the viability and proliferation of TM3 cells, which might be related to ferroptosis-mediated inflammation. Compared with other RCD pathways, ferroptosis, a programmed non apoptotic cell death, is driven by lethal lipid peroxidation and iron homeostasis imbalance [30]. The iron homeostasis imbalance may be partially due to NCOA4-mediated ferritinophagy, which releases iron stored in ferritin into the labile iron pool [31,32]. We found that CCC can cause the accumulation of lipid peroxidation products, increase mitochondrial ROS level, and interfere with the expression of FTL and NOCA4, leading to an imbalance of iron homeostasis in TM3 cells. Fer-1, an inhibitor of ferroptosis, could reverse the damage of CCC to TM3 cells, indicating that ferroptosis might be one of the main contributors to CCC-induced inhibition of TM3 cell viability and proliferation.

Glutathione peroxidase 4 (GPX4) is a lipid repair enzyme that utilizes glutathione (GSH) as a cofactor to convert harmful lipid hydroperoxides into nontoxic lipid alcohols [33,34]. The content of GSH is regulated by xCT (also known as SLC7A11), which is a component of the xc system amino acid antiporter. It exports intracellular glutamate in exchange for extracellular cysteine, which is a synthetic material of GSH [35]. The GSH content is also related to thioredoxin-interacting protein (TXNIP). TXNIP is an α-arrestin family member regulating cellular oxidative stress by controlling thioredoxin (TRX) reducing capacity [36]. TXNIP can function as a potent negative regulator of glucose uptake and aerobic glycolysis. Lowering TXNIP would inhibit aerobic glycolysis and decrease substrate flux through the pentose phosphate pathway to produce less NADPH and GSH [37]. In addition, the functional defects of TXNIP may have fatal consequences on cells and accelerate destructive inflammation. Ji et al. [27] showed that the loss of TXNIP caused by oxidative stress reduced retinal pigment epithelium (RPE) cell proliferation. And the depletion of TXNIP in ARPE-19 cells induced autophagy through p53-mediated AMPK activation. Additionally, unlike GPX4, FACL4, also known as ACSL4 (Acyl-CoA Synthetase Long-Chain Family Member 4), can positively regulate ferroptosis [38]. However, in the present study, CCC exposure did not alter the levels of xCT, FACL4, and p-p53, but TXNIP expression was rapidly downregulated, and GPX4 expression increased in TM3 cells. These results indicated that in addition to inducing lipid peroxidation, CCC might also reduce GSH content by inhibiting TXNIP expression and stimulate GPX4 production to cope with ferroptosis. Moreover, Fer-1 could only partially improve GPX4 level and proliferation inhibition, possibly due to its inability to improve TXNIP expression.

In addition to discovering that CCC induced ferroptosis in TM3 cells, we also confirmed that cell apoptosis and pyroptosis were related to CCC exposure. The activation of inflammasomes can trigger inflammation and innate immune responses by releasing cytokines and inducing pyroptosis [39]. Previous research has found that GPX4 expression and lipid peroxidation were involved in GSDMD-mediated pyroptosis in sepsis [40]. By comparison, we analyzed the effects of Fer-1 and ZVF on lipid peroxidation- and Caspase-mediated RCD. We observed that Fer-1 could not prevent the activation of Caspase3 after CCC exposure, indicating that Fer-1 could not inhibit apoptosis. This was consistent with the research by Vats et al. [13], who found that Fer-1 was unable to prevent Caspase3 activation in primary human epidermal keratinocytes after exposure to ultraviolet B radiation. However, although ZVF inhibited all Caspase activation, such as non-inflammatory responses triggered by apoptosis and inflammatory responses triggered by pyroptosis, we found that it did not significantly reduce lipid peroxidation like Fer-1. Both Fer-1 and ZVF inhibited the activation of inflammasome-dependent Caspase1 triggered by CCC, while ZVF could not inhibit the release of IL-1β and damage-associated molecular pattern (DAMP) marker HMGB1, suggesting that ferroptosis might be the main pathway leading to inflammation.

FTL is a key regulatory factor in maintaining intracellular iron homeostasis events and plays a role in cell protection, proliferation, and metabolism [41]. According to reports, FTL can ameliorate LPS-induced production of inflammatory cytokines and ROS, as well as improve intracellular labile iron pool [42]. It can also prevent inflammation and organ damage caused by sepsis [23]. In our study, overexpression of FTL to some extent reduced the sensitivity of cells to CCC, weakening the damage of CCC to the proliferation and viability of TM3 cells. In FTL overexpressing cells treated with CCC, the expression of NOCA4, TXNIP and GPX4 was not significantly impaired, and NLRP3-mediated inflammation was weakened. Additionally, CCC could still cause altered expression of NOCA4 nad TXNIP in FTL knockout cells, as well as increased expression of NLRP3, cleaved-Caspase1, and GSDMD-N. But, regardless of the presence of CCC, FTL knockout significantly inhibited IL-1β. Therefore, knocking down FTL might contribute to impaired cell-mediated immunity. In fact, moderate inflammation is a reaction of the immune system induced in response to infection or tissue injury, which is essential for efficient host defense and tissue repair [43]. Hence, further work is needed to elucidate the relationship between lipid peroxidation, inflammasome activation, and ferroptosis-induced inflammation.

## 5. Conclusions

In conclusion, this study found that CCC could induce lipid peroxidation and elevated mitochondrial ROS level and iron homeostasis imbalance in TM3 cells, leading to ferroptosis. Although some TM3 cell death after exposure to CCC might be related to apoptosis and pyroptosis, ferroptosis might be a critical non-apoptotic RCD mode responsible for the CCC-driven Leydig cell inflammation. The abnormal levels of GPX4 might be associated with the susceptibility of TM3 to ferroptosis. The ferroptosis inhibitor Fer-1 could alleviate the ferroptosis and inflammatory response caused by CCC. In addition, overexpression of FTL to a certain extent repaired CCC-induced weakened viability and proliferation of TM3 cells by improving ferroptosis-mediated inflammation, but FTL deletion might cause immunosuppression. Therefore, ferroptosis might be involved in the mechanism of Leydig cell growth disorders caused by CCC.

## Figures and Tables

**Table 1:** Construction framework of FTL-shRNA lentiviral vectors.

NO.	5′	Stemp	Loop	Stemp	3′
FTL-shRNA-A-1	CCGG	GAAGCCATCTCAAGATGAATG	CTCGAG	CATTCATCTTGAGATGGCTTC	TTTTTTG
FTL-shRNA-A-2	aattcaaaaaa	GAAGCCATCTCAAGATGAATG	CTCGAG	CATTCATCTTGAGATGGCTTC	
FTL-shRNA-B-1	CCGG	CCATGGAGAAGAACCTGAATC	CTCGAG	GATTCAGGTTCTTCTCCATGG	TTTTTTG
FTL-shRNA-B-2	aattcaaaaaa	CCATGGAGAAGAACCTGAATC	CTCGAG	GATTCAGGTTCTTCTCCATGG	
FTL-shRNA-C-1	CCGG	CTCTGGGCGAGTATCTCTTTG	CTCGAG	CAAAGAGATACTCGCCCAGAG	TTTTTTG
FTL-shRNA-C-2	aattcaaaaaa	CTCTGGGCGAGTATCTCTTTG	CTCGAG	CAAAGAGATACTCGCCCAGAG	
FTL-shRNA-NC-1	CCGG	TTCTCCGAACGTGTCACGT	TTCAAGAGA	ACGTGACACGTTCGGAGAA	TTTTTTG
FTL-shRNA-NC-2	aattcaaaaaa	TTCTCCGAACGTGTCACGT	TCTCTTGAA	ACGTGACACGTTCGGAGAA	

## Data Availability

The raw data supporting the conclusions of this article will be made available by the authors on request.

## Supplementary Materials

The following supporting information can be downloaded at: https://www.mdpi.com/article/10.3390/cells13110979/s1, Figure S1: CCC did not alter the expression of P53 and p-P53 in TM3 cells; Figure S2: CCC did not cause necroptosis in TM3 cells.

## Abbreviations

CCC	Chlormequat chloride
FTL	Ferritin light chain
RCD	Regulated cell death
IL-1β	Interleukin-1β
RIPK3	Receptor interacting protein kinase 3
p-RIP3	Phospho- RIPK3
RIPK1	Receptor interacting protein kinase 1
MLKL	Mixed-lineage kinase domain-like protein
p-MLKL	Phospho-MLKL
FTH	Ferritin heavy chain
PCRC	Peking Union Medical College
PCR	Polymerase chain reaction
MOI	Multiplicity of infection
GFP	Green fluorescence protein
CCK-8	Cell Counting Kit-8
MitoSOX	Mitochondrial superoxide
ROS	Reactive oxygen species
MFI	Mean fluorescence intensity
PBS	Phosphate-buffered saline
CFSE	Carboxyfluorescein succinimidyl ester
GAPDH	Glyceraldehyde-3-phosphate dehydrogenase
Caspase	Cysteinyl aspartate specific proteinase
p-P53	Phospho-P53
NLRP3	NOD-, LRR-, and pyrin-domain-containing protein 3
GPX4	Glutathione peroxidase 4
HMGB1	High-mobility group box-1 protein
TXNIP	Thioredoxin-interacting protein
TfR	Transferrin receptor
FACL4	Fatty acid-CoA ligase 4
xCT	Solute carrier family 7 member 11
GSDMD	Gasdermin-D
GSDMD-N	GSDMD N-Terminal
NOCA4	Nuclear receptor coactivator 4
TBST	Tris-Buffered Saline with Tween^®^ 20
DDR	DNA damage response
DAMP	Damage-associated molecular pattern
ANOVA	Analysis of variance
ZVF	Z-VAD-FMK
Fer-1	Ferrostatin-1
NS	No statistical significance
LPS	Lipopolysaccharides
GSH	Glutathione
TRX	Thioredoxin
NADPH	Nicotinamide adenine dinucleotide phosphate
RPE	Retinal pigment epithelium
AMPK	Adenosine 5′-monophosphate (AMP)-activated protein kinase
