# Supplementary material for: Chlormequat Chloride Inhibits TM3 Leydig Cell Growth via Ferroptosis-Initiated Inflammation
Source: Cells. 2024 Jun 5;13(11):979. doi: 10.3390/cells13110979 (PMC11171675; doi:10.3390/cells13110979)
Supplement: Supplementary file 1 [file cells-13-00979-s001.zip › cells-3000960-supplementary.pdf]

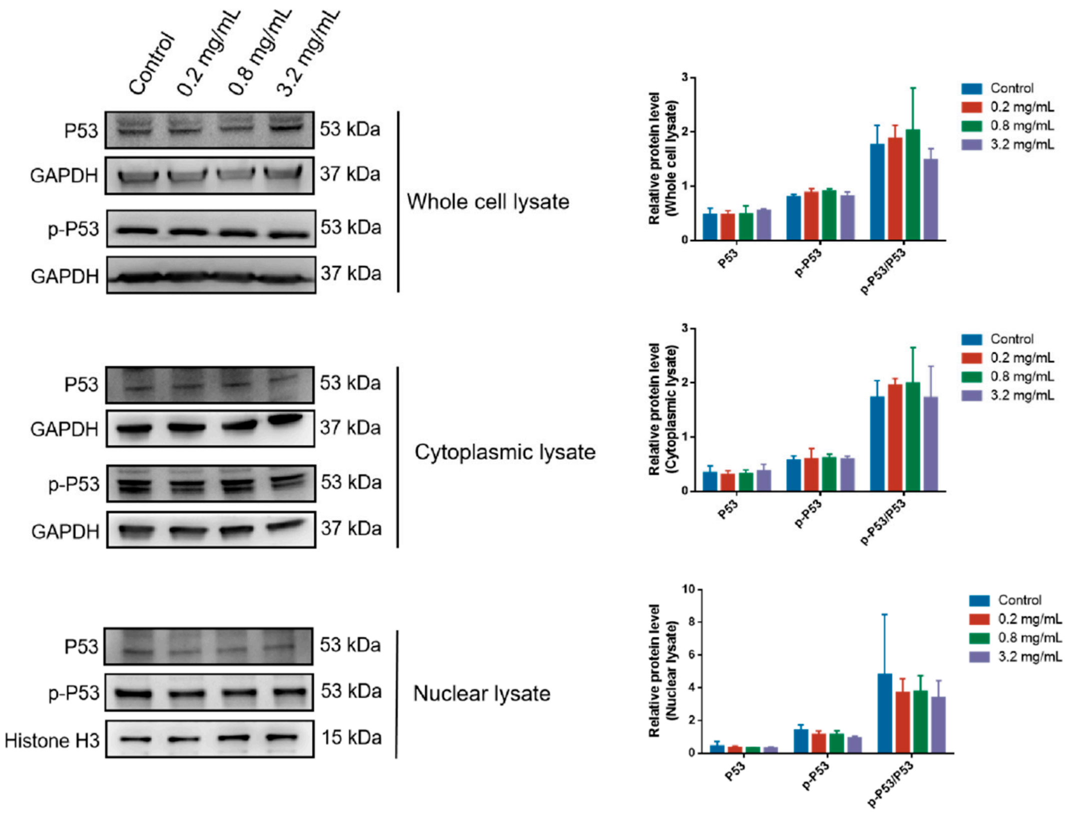

Figure S1 CCC did not alter the expression of P53 and p-P53 in TM3 cells. Representative Western blots of P53 and p-P53 in the nucleus, cytoplasm and whole cell lysate, as well as semi-quantitative analysis of protein expression, calculating the relative expression of each protein using target protein/internal reference protein; n=3. All data were expressed as mean ± SD. Statistical significance was tested by one-way ANOVA, $^{*}p<0.05$ vs. the control group, and $^{**}p<0.01$ vs. the control group. ANOVA, analysis of variance.

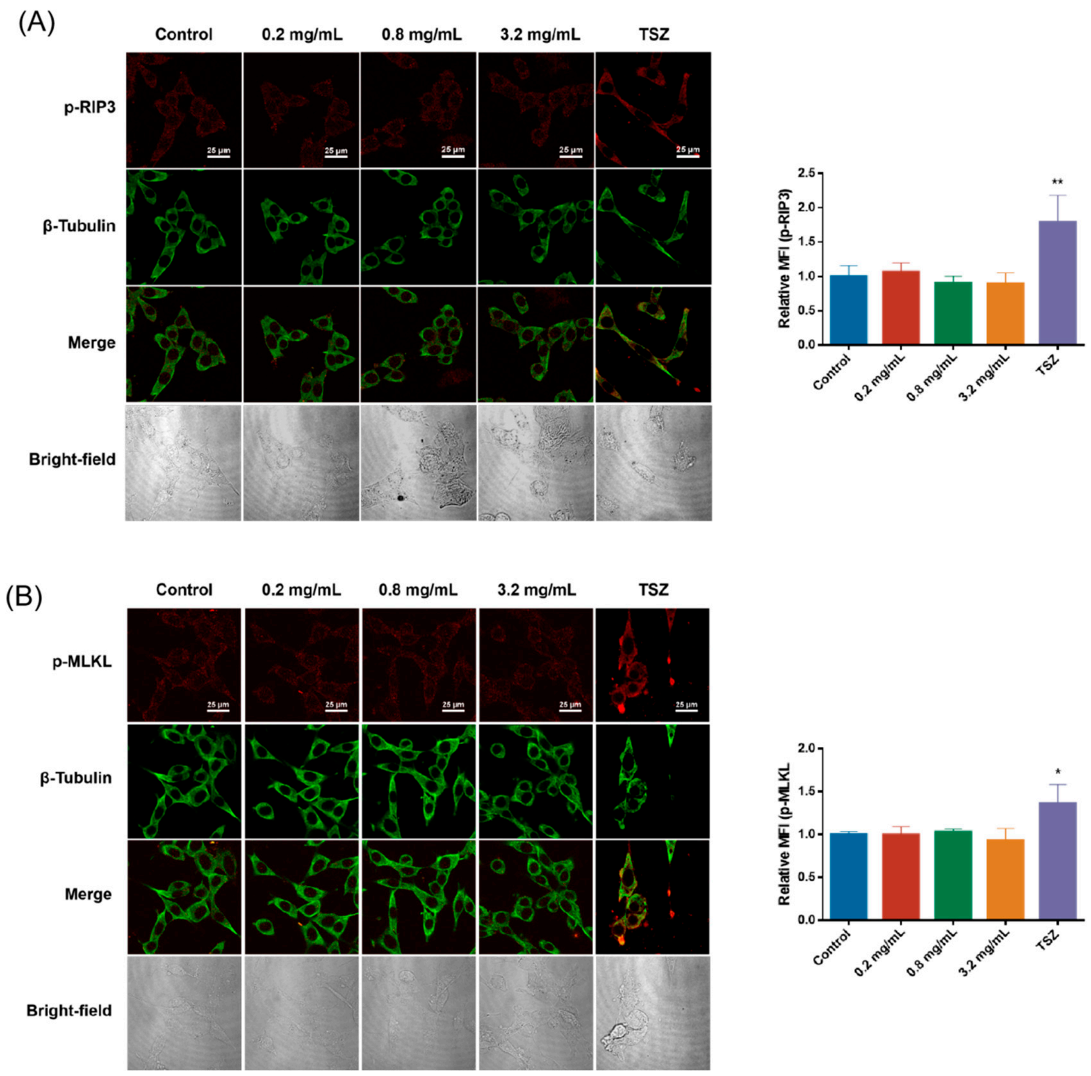

Figure S2 CCC did not cause necroptosis in TM3 cells. Representative fluorescence images of TM3 cells stained with p-MLKL and p-RIP3 (red-p-MLKL/p-RIP3, green-β-Tubulin), and their relative MFI (100×, scale bar = 25 μm). Necroptosis inducer TSZ was used to treat TM3 cells for 12 hours as a positive control for p-MLKL and p-IRF3. All data were expressed as mean ± SD (n=3). Statistical significance was tested by one-way ANOVA, $^{*}p<0.05$ vs. the control group. ANOVA, analysis of variance.
